# Multi-omics integration reveals macrophage polarization and ferroptosis as key mechanisms underlying kaempferol’s therapeutic efficacy in peripheral artery disease

**DOI:** 10.3389/fimmu.2026.1733284

**Published:** 2026-09-03

**Authors:** Zi-jun Qiao, Miao-miao Wang, Wang-sheng Luo, Bo-lan Yu, Tao Liu, Ping Zhu, Can Hu, Ji Li, Zhi-qiang Liang, Jian Chen

**Affiliations:** 1 Guangdong Cardiovascular Institute, Guangdong Provincial People's Hospital (Guangdong Academy of Medical Sciences), Southern Medical University, Guangzhou, Guangdong, China; 2 Guangdong Provincial Key Laboratory of Pathogenesis, Targeted Prevention and Treatment of Heart Disease,Guangzhou Key Laboratory of Cardiac Pathogenesis and Prevention, Guangzhou, Guangdong, China; 3 Institute of Vascular Anomalies, Shanghai TCM-Integrated Hospital, Shanghai University of Traditional Chinese Medicine, Shanghai, China; 4 Department of Cardiology, the First Affiliated Hospital of University of South China, Hengyang, China; 5 School of Medicine South China University of Technology, Guangdong Provincial People's Hospital, Guangdong Academy of Medical Sciences, Southern Medical University, Guangzhou, Guangdong, China; 6 Department of Hepatobiliary Surgery, Shandong Provincial Hospital Heze Branch, Heze, China; 7 Guangzhou Xiangxue Pharmaceutical Co., Ltd, Guangzhou, China

**Keywords:** macrophage, natural products, network pharmacology, omics, peripheral artery disease

## Abstract

Peripheral artery disease (PAD) is a chronic ischemic condition characterized by vascular occlusion and metabolic-immune dysregulation. To elucidate the molecular mechanisms underlying PAD and the therapeutic effects of Kaempferol, we integrated bulk transcriptomics, single-cell RNA sequencing, network pharmacology, and experimental validation. Transcriptomic analysis identified 1,144 differentially expressed genes in PAD, highlighting extensive transcriptional remodeling. WGCNA revealed distinct gene modules significantly correlated with PAD, suggesting key networks associated with vascular inflammation and immune dysregulation. Network pharmacology identified kaempferol as the core active component of Ruan Jian Qing Mai formula that is clinically effective in treating PAD, targeting 87 PAD-related genes enriched in immune and inflammatory pathways, predominantly expressed in macrophages. Integration of kaempferol-associated targets from RNA-seq and multi-database prediction uncovered 70 overlapping targets implicated in macrophage activation, lipid metabolism, and atherosclerosis signaling. CIBERSORTx and single-cell analysis further pinpointed macrophages as major cellular mediators, with kaempferol-target genes (e.g., TNF, SYK, LCK, IL4I1) specifically enriched in macrophages. Macrophages were stratified into high-inflammatory and low-inflammatory subsets; PAD lesions exhibited prominent enrichment of high-inflammatory macrophages, which concurrently displayed elevated inflammatory signatures and enhanced ferroptosis transcriptional activity, and represented the primary cellular population responsive to kaempferol. In silico gene knockout analysis further verified that SYK and SNX5 serve as core immune signaling regulators in macrophages, while TNF and LCK lack macrophage-specific immune regulatory functions. Functional experiments confirmed that kaempferol significantly suppressed LPS-induced M1 macrophage polarization and inflammatory cytokine expression in RAW264.7 cells. Ferroptosis scoring revealed enhanced ferroptosis susceptibility in PAD macrophages, with kaempferol-target genes correlating positively with ferroptosis drivers. Molecular docking further validated strong binding affinities between kaempferol and key macrophage-related targets (IL4I1, LCK, TNF, SYK). Using a zebrafish vascular regeneration injury model, we further verified that high-dose kaempferol rescues impaired angiogenesis under vascular ischemic damage. Collectively, these multi-omics and experimental findings uncover macrophage polarization and ferroptosis modulation as central mechanisms mediating kaempferol’s therapeutic efficacy in PAD, providing mechanistic insights and potential molecular targets for precision intervention.

## Introduction

Peripheral artery disease (PAD) is a prevalent circulatory disorder characterized by the narrowing of arteries, leading to reduced blood flow to the limbs (1). This condition is often associated with significant morbidity, impacting patients’ quality of life and increasing healthcare costs across systems (2). Despite the availability of various treatment modalities, including lifestyle modifications, pharmacotherapy, and surgical interventions, many patients continue to experience limitations due to insufficient efficacy and the inability of current therapies to address the underlying pathophysiological mechanisms of PAD (3). The urgency of improving therapeutic strategies for PAD is underscored by its association with increased cardiovascular events, highlighting the need for novel interventions that can effectively target the disease process (4).

Recent studies have begun to elucidate the molecular underpinnings of PAD, revealing critical insights into its complex pathophysiology. The role of inflammation and immune cell dynamics has gained particular attention, as inflammatory processes are known to contribute to the progression of atherosclerosis and subsequent PAD development (5). Moreover, the identification of specific biomarkers associated with PAD has opened new avenues for understanding disease mechanisms and developing targeted therapies (6). However, a comprehensive understanding of how key molecular pathways interact across different cellular compartments and contribute to PAD progression remains limited. Furthermore, the therapeutic potential of natural compounds targeting these dysregulated pathways, particularly macrophage-centric mechanisms, warrants deeper investigation.

This study aims to bridge these gaps by exploring the therapeutic potential of kaempferol, a natural flavonoid recognized for its anti-inflammatory and antioxidant properties (7). Previous research has suggested that kaempferol may exert beneficial effects in cardiovascular diseases, including its ability to modulate inflammatory responses and oxidative stress (8). The integration of bioinformatics methodologies, including differential expression analysis and weighted gene co-expression network analysis (WGCNA), presents a powerful approach to identify and characterize the genetic alterations associated with PAD, as well as the potential therapeutic targets of kaempferol (9).

To address these gaps and provide a systems-level view, this study employs an integrated multi-omics bioinformatics framework. We analyze bulk transcriptomic data from human PAD arteries to identify core disease-associated pathways and differentially expressed genes. Complementing this, we utilize scRNA-seq data from PAD to dissect cellular heterogeneity and identify cell-type-specific dysregulation, with a focus on macrophage populations implicated in PAD pathogenesis. To elucidate the potential therapeutic mechanisms of kaempferol, we integrate its transcriptional targets and predicted targets from public databases. This methodology facilitates the identification of key molecular pathways involved in PAD and enables the characterization of immune cell profiles, particularly focusing on macrophage polarization and inflammatory mechanisms (10). By systematically intersecting these datasets and applying advanced network analyses, we aim to move beyond general therapeutic descriptions and specifically pinpoint the critical crosstalk regulation between macrophage polarization and ferroptosis. This multi-omics integration reveals the core mechanism by which kaempferol modulates the immuno-metabolic microenvironment in PAD.

In summary, this research seeks to investigate the intricate relationship between kaempferol and PAD by leveraging advanced bioinformatics tools to uncover new insights into disease mechanisms and therapeutic targets. By addressing the existing gaps in our understanding of PAD pathophysiology and exploring the potential of kaempferol as a therapeutic agent, this study aims to contribute to the development of more effective treatment options for patients suffering from this debilitating condition.

## Methods

### Data collection from the GEO database and differential expression

For the bulk transcriptomic analysis, to eliminate the profound spatial transcriptomic noise associated with different vascular beds, the 14 PAD and 11 Control samples from the GSE100927 dataset were exclusively selected from the infra-popliteal artery cohort, representing the distal pathology most intimately associated with critical limb ischemia. Furthermore, the single-nuclei RNA sequencing dataset GSE233882 comprised 32 total biological subjects (20 PAD, 12 Control). In the bioinformatic pipeline, these were analyzed as 3 PAD and 2 non-PAD samples, which accurately reflects the pooled technical sequencing libraries utilized by the original investigators to maximize capture efficiency and mitigate inter-patient batch effects. RNA-seq data for kaempferol-treated mice (GSE145665) was analyzed to identify drug-responsive genes. Differential expression analysis was performed using Sangerbox 3.0 (http://vip.sangerbox.com/home.html) and the DESeq2 R package. Genes with an absolute fold change ≥ 1.5 and FDR < 0.05 were defined as differentially expressed genes (DEGs). Principal component analysis (PCA) was utilized to visualize sample clustering.

### Weighted gene co-expression network analysis

Weighted gene co-expression network analysis (WGCNA) was performed on the GSE100927 dataset to identify clinically relevant gene modules (11). A soft-thresholding power was determined to ensure a scale-free network topology, and the adjacency matrix was transformed into a topological overlap matrix (TOM) for hierarchical clustering. Co-expressed gene modules were identified and merged based on similarity, followed by the analysis of module-trait relationships to pinpoint key disease-associated modules and hub genes.

### Identification of active components and integrative network pharmacology

To identify the core bioactive ingredients of the Ruan Jian Qing Mai (RJQM) formula, we first screened its absorbed components using the HERB (12), NetInfer (13), and the SwissTargetPrediction (14) databases. Kaempferol was identified as a key candidate monomer based on its network degree centrality. Subsequently, to comprehensively map kaempferol’s specific therapeutic targets, we integrated predictions from SwissTargetPrediction with experimental transcriptomic data from kaempferol-treated mice (GSE145665). Differential expression analysis was performed using DESeq2 (|fold change| ≥ 1.5, FDR < 0.05) to validate drug-responsive genes. These consolidated kaempferol targets were then intersected with PAD-specific DEGs and WGCNA modules to pinpoint disease-relevant targets. Finally, the core overlapping genes were subjected to GO and KEGG pathway enrichment analyses using the STRING database (v12.0), and a Protein–protein interaction (PPI) network was constructed and visualized using Cytoscape (v3.10.0) to elucidate the regulatory mechanisms.

### Immune cell profiling and macrophage insights into kaempferol’s targets in PAD

To evaluate the impact of the immune microenvironment on the progression of PAD, we conducted a comprehensive analysis of immune infiltration patterns in both PAD and control groups utilizing the CIBERSORTx tool (https://cibersortx.stanford.edu/). Orthogonal Partial Least Squares Discriminant Analysis (OPLS-DA) was performed to classify the infiltrating immune cell profiles between PAD and control groups. The cell-specific gene coexpression networks were collected from the Immuno-Navigator database with Networkanalyst online tools (15). Furthermore, to examine the relationship between key hub genes and immune cells exhibiting differential infiltration, we implemented a Pearson correlation coefficient analysis.

### Analysis of the heterogeneity in PAD single-cell transcriptomes

Single-cell RNA-seq data from skeletal muscle (GSE233882, 3 PAD vs. 2 Controls) were analyzed to investigate cellular heterogeneity. Quality control was strictly enforced by excluding cells with <200 or >5,000 genes or >10% mitochondrial content. Batch effects were corrected using the Harmony package (v0.1.0) (16), followed by UMAP dimensionality reduction and clustering to visualize cell type distributions and target expression. Furthermore, hierarchical directed WGCNA (hdWGCNA) was employed to construct co-expression networks specifically focused on macrophage polarization dynamics within the PAD microenvironment. scTenifoldKnk performed virtual knockout of each target gene on all macrophages using 2,000 genes. Differential regulatory genes were identified at p.adj < 0.05. GO Biological Process enrichment (clusterProfiler enrichGO, BH correction, p < 0.1) was performed on significant differential regulatory genes, with a top-50 fallback when fewer than 5 genes were significant at p.adj < 0.10.

### Ferroptosis driver scores and kaempferol-macrophage-targeted gene correlations in PAD

To systematically quantify ferroptosis activity, verified ferroptosis-related gene sets were retrieved from the FerrDb V2 database (17) and categorized into three groups: drivers (FD), suppressors (FS), and markers (FM). Single-cell gene signature scores were calculated using the UCell R package, which employs the Mann-Whitney U statistic to compute rank-based enrichment scores robust to dataset size and dropout events. Subsequently, Pearson correlation analysis was performed to evaluate the relationship between ferroptosis scores, macrophage infiltration, and kaempferol target expression.

### Cell culture

The Mouse Monocytic Macrophage Leukemia Cell Line (RAW264.7) was obtained from the American Type Culture Collection (ATCC, USA) and was cultured in Dulbecco’s Modified Eagle Medium (Gibco, 11995073), which was enriched with 10% Fetal Bovine Serum (FBS; Gibco, 10099-141C) and 1% Penicillin-Streptomycin Solution (Sigma, V900929). To simulate the localized, hyper-inflammatory immune microenvironment characteristic of severe peripheral artery disease *in vitro*, RAW264.7 macrophages were stimulated with Lipopolysaccharide (LPS) to induce a classical pro-inflammatory M1-like activation state. Simultaneously, the cells were treated with kaempferol at a concentration of 50 μM. This specific concentration was selected based on previous literature, which reported optimal therapeutic efficacy and cellular safety at this dosage (18). RAW264.7 cells were pre-treated with kaempferol (dissolved in DMSO) for 2 hours prior to stimulation with LPS. The cells were subsequently exposed to LPS for 6 hours for RNA extraction and qPCR analysis, and 24 hours for supernatant harvesting and Nitric Oxide quantification. Cells were stained with PE-anti-CD86 (#374206, Biolegend), and analyzed using flow cytometry. To eliminate solvent artifact bias, all baseline control groups utilized in these experiments represent a vehicle control, consisting of culture medium supplemented with an equivalent concentration of DMSO (≤0.1% v/v). All cell lines were maintained in a controlled environment at 37 °C with 5% CO2 in a humidified atmosphere.

### NO assay

After administering RAW264.7 cells, the supernatant was collected and centrifuged at 3000 rpm for 10 minutes to clarify the sample. Following this, 50 µl of the clarified supernatant was transferred into a 96-well plate. According to the protocol outlined in the NO assay kit (Beyotime, S0021), reagents 1 and 2 were added to the wells. The optical density (OD) was then measured at a wavelength of 450 nm using a microplate reader to assess the results.

### Validation of M1 and inflammation genes via RT-qPCR

Total RNA was extracted using TRIzol reagent (Invitrogen), and cDNA was synthesized using a cDNA Synthesis SuperMix. RT-qPCR was performed to quantify the expression of M1 polarization and inflammatory genes, normalized to GAPDH. The specific primer sequences are listed in **Supplementary Table 1**. Relative gene expression levels were calculated using the 2^−ΔΔ^CT method.

### Molecular docking

To elucidate the binding modes between kaempferol and its targets, molecular docking was performed using a blind docking strategy via the CB-Dock2 server (19). The 3D crystal structures of the target proteins were retrieved from the RCSB PDB and AlphaFold Database, specifically: TNF (PDB: 6X86), SNX5 (PDB: 5TGI), LCK (PDB: 2OF2), SYK (PDB: 6ZCX), RTP4 (AF-Q96DX8-F1), IL4I1 (AF-Q96RQ9-F1), HHEX (AF-Q03014-F1), and KCNE3 (AF-Q9Y6H6-F1). The 3D structure of kaempferol (PubChem CID: 5280863) was obtained in SDF format. To validate the docking protocol, the native ligands co-crystallized within the structures of TNF, SYK, and LCK were extracted and re-docked as positive controls. Unlike manual grid generation, CB-Dock2 integrates CurPocket for curvature-based cavity detection to automatically identify binding pockets and define grid box coordinates, followed by AutoDock Vina for scoring. The conformation with the lowest binding energy (Vina score) in the largest cavity was selected, and interaction types were analyzed based on standards outlined in previous studies (20, 21).

### Zebrafish vascular regeneration injury model

Egg water was prepared by diluting sea salt stock and methylene blue in deionized water, then maintained at 28 °C for zebrafish embryo culture. Wild-type embryos were distributed into six-well plates and assigned to four groups: blank control, 160 nM VRI injury model group, and two kaempferol treatment groups (10 μM/20 μM). For model construction: embryos were incubated with 160 nM VRI for 24 h to trigger vascular damage. The VRI solution was then discarded, and embryos were cultured in fresh egg water for another 24 h. For drug groups, post-VRI incubation, embryos were cultured with two concentrations of kaempferol for the subsequent 24 h. Prior to observation, embryos were anesthetized with 1% ethanol egg water and mounted on glass slides. Fluorescence microscopy was used to image intersegmental vessels (ISVs). A unified scoring standard was adopted to quantify vascular integrity: ISV score = intact vessel count × 1 + incomplete vessel count × 0.5. All embryos were humanely euthanized via overdose of 20% ethanol combined with ice-water hypoxia after imaging acquisition.

### Scoring and statistical analysis

UCell scoring was applied to all macrophages for gene sets: inflammatory, ferroptosis driver, Kaempferol-up (203 genes), and Kaempferol-down (294 genes). Composite scores were computed as up minus down (Kaempferol). Macrophages were split by median inflammatory score into High-Inflammatory and Low-Inflammatory groups. Wilcoxon rank-sum tests with Benjamini-Hochberg correction compared scores between groups. Pearson and Spearman correlations assessed continuous relationships. Chi-squared test evaluated condition-group association.

## Results

### Identification of differentially expressed genes in peripheral artery disease

**Figure 1:**
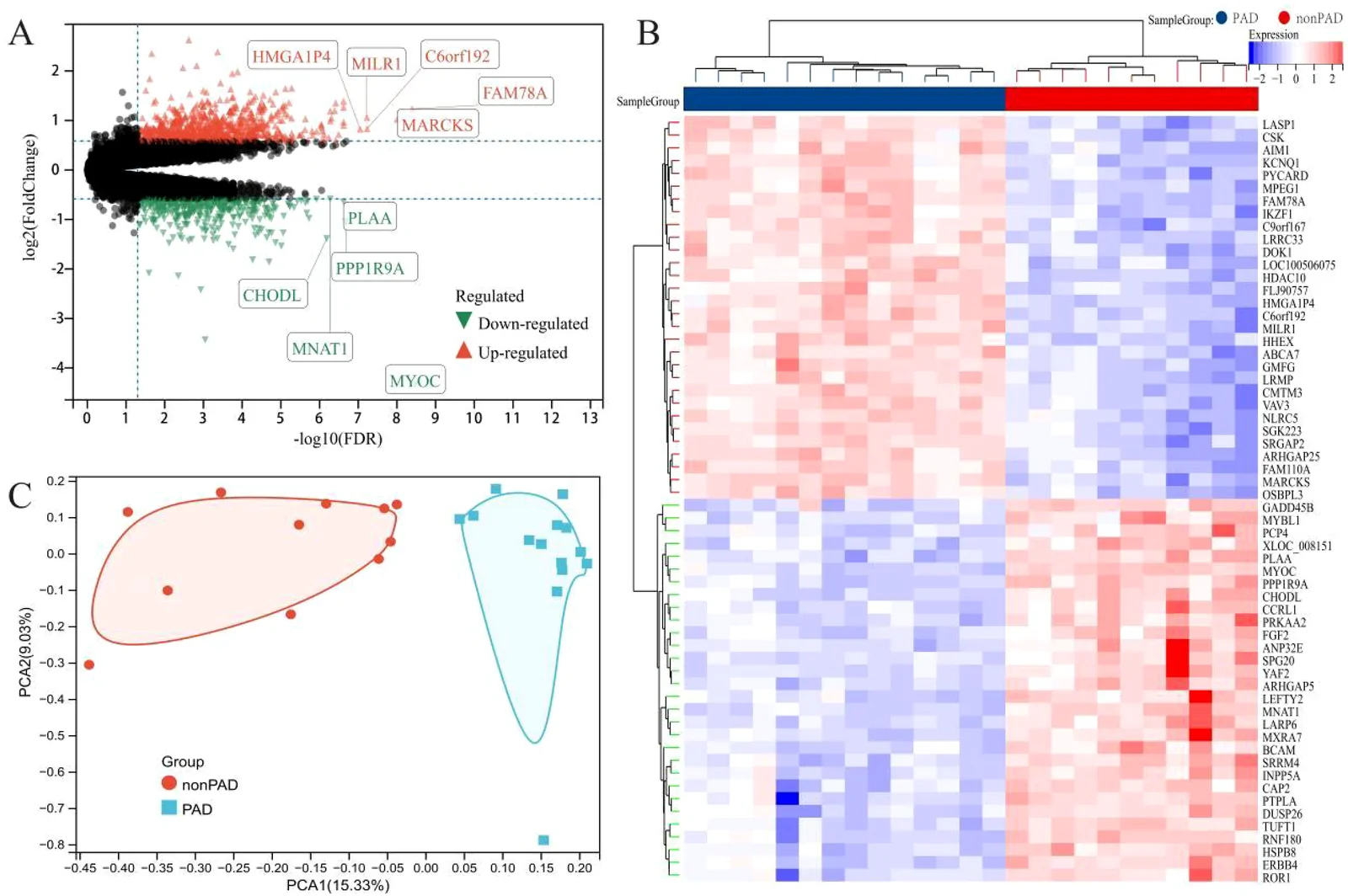
Identification of differentially expressed genes in peripheral artery disease. **(A)** Volcano plot of differentially expressed genes between PAD and nonPAD groups. **(B)** Heatmap of hierarchical clustering showing the expression patterns of top 30 genes across PAD and nonPAD groups. **(C)** Principal component analysis (PCA) plot of PAD and nonPAD groups. The x-axis represents PCA1, which explains 15.33% of the variance, and the y-axis represents PCA2, which explains 9.03% of the variance.

Analysis of the GSE100927 arterial transcriptome dataset revealed profound gene expression alterations in PAD patients compared to healthy controls. We identified 1144 differentially expressed genes (|fold change| ≥ 1.5 and FDR value < 0.05) in PAD, of which 728 genes were up-regulated and 416 genes were down-regulated. In Genes such as HMGA1P4, MARCKS, and MYOC were prominently up-regulated, while PLAA, PPP1R9A, and CHODL were down-regulated (**Figure 1A**). **Figure 1B** presents a heatmap of hierarchical clustering, showing the expression patterns of top 30 genes across the two sample groups. The heatmap demonstrates distinct expression profiles between PAD (blue) and nonPAD (red) groups, with genes such as LASPI, CSK, and AIM1 showing higher expression in the nonPAD group, while genes like MYOC, PPP1R9A, and CHODL exhibit higher expression in the PAD group. Principal component analysis (PCA) further confirmed distinct transcriptomic profiles for the PAD and control groups, primarily along the first principal component (PCA1, 15.33% variance; **Figure 1C**). This widespread dysregulation underscores the molecular complexity of PAD and highlights potential disease drivers and pathways for therapeutic intervention.

### WGCNA analysis reveals distinct gene modules and their associations with control and PAD groups

**Figure 2:**
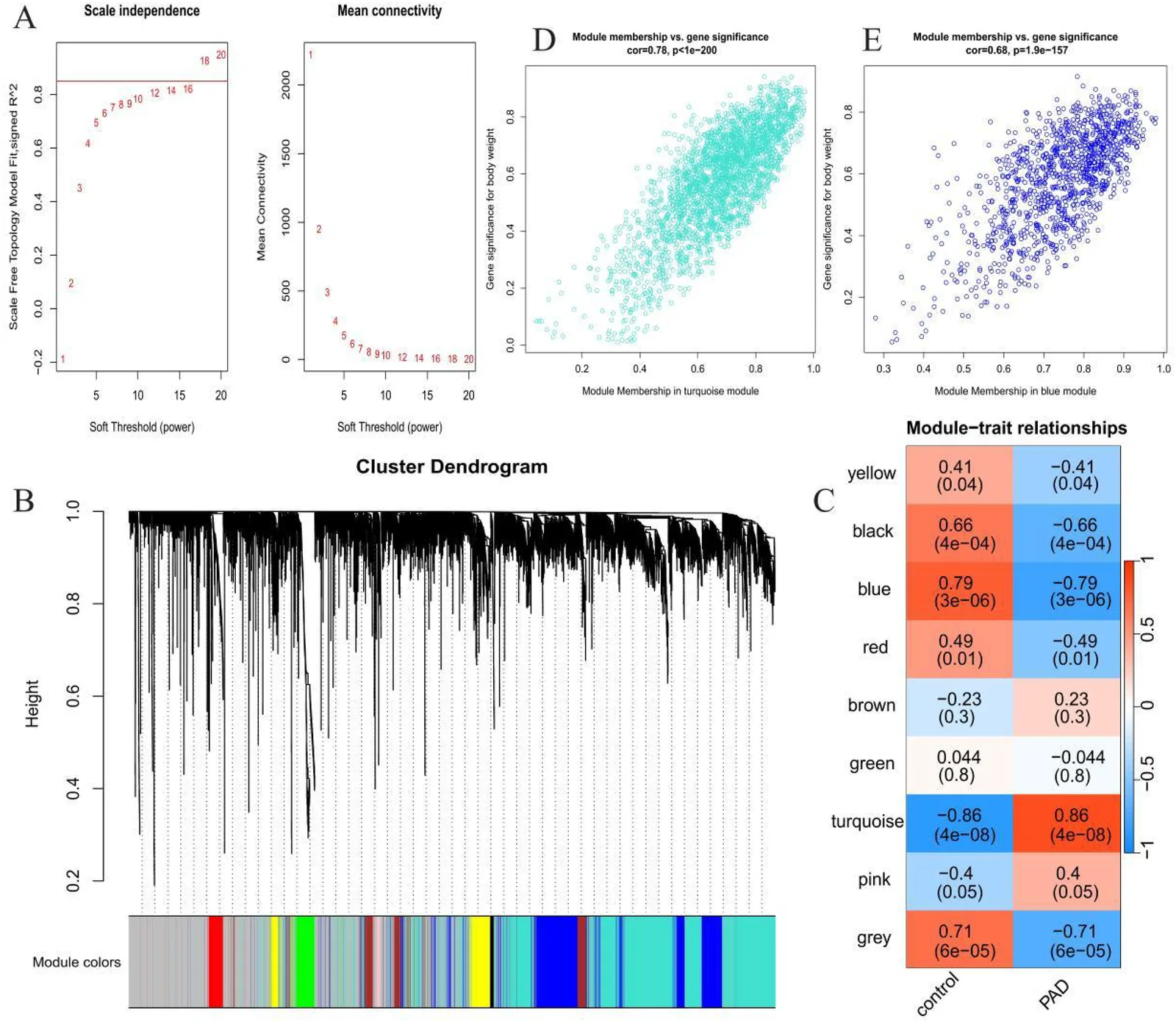
WGCNA analysis reveals distinct gene modules and their associations with control and PAD groups. **(A)** Selection of the soft threshold power for WGCNA. The left panel shows the scale-free topology fit index (R²) as a function of the soft threshold power, while the right panel displays the mean connectivity. **(B)** Hierarchical clustering dendrogram of gene expression profiles. Genes are grouped into distinct modules, each represented by a unique color at the bottom of the dendrogram. **(C)** Heatmap of module-trait relationships. The heatmap shows the correlation coefficients between each module and the control/PAD groups, with the corresponding p-values in parentheses. Scatter plot of module membership vs. gene significance for PAD in the turquoise **(D)** and blue **(E)** modules.

Then, we employed WGCNA to identify gene modules and their associations with control and PAD groups. **Figure 2A** illustrates the power of 18 was chosen based on the scale-free topology fit index and mean connectivity. The hierarchical clustering dendrogram (**Figure 2B**) shows the gene expression profiles grouped into distinct modules. This analysis revealed several modules significantly correlated with disease state (**Figure 2C**). Most notably, the turquoise module (2,505 genes) exhibited a strong positive correlation with PAD (r=0.86, p=4e-08), while the blue module (1,154 genes) showed a strong negative correlation (r=-0.79, p=3e-06). **Figure 2D, E** show scatter plots illustrating the correlation between module membership and gene significance for body weight in the turquoise and blue modules. The significant association of these large modules with PAD suggests they encompass key biological processes driving disease progression or protective responses, respectively.

### Network pharmacology analysis identified kaempferol as a core active component of RJQM in the treatment of PAD

**Figure 3:**
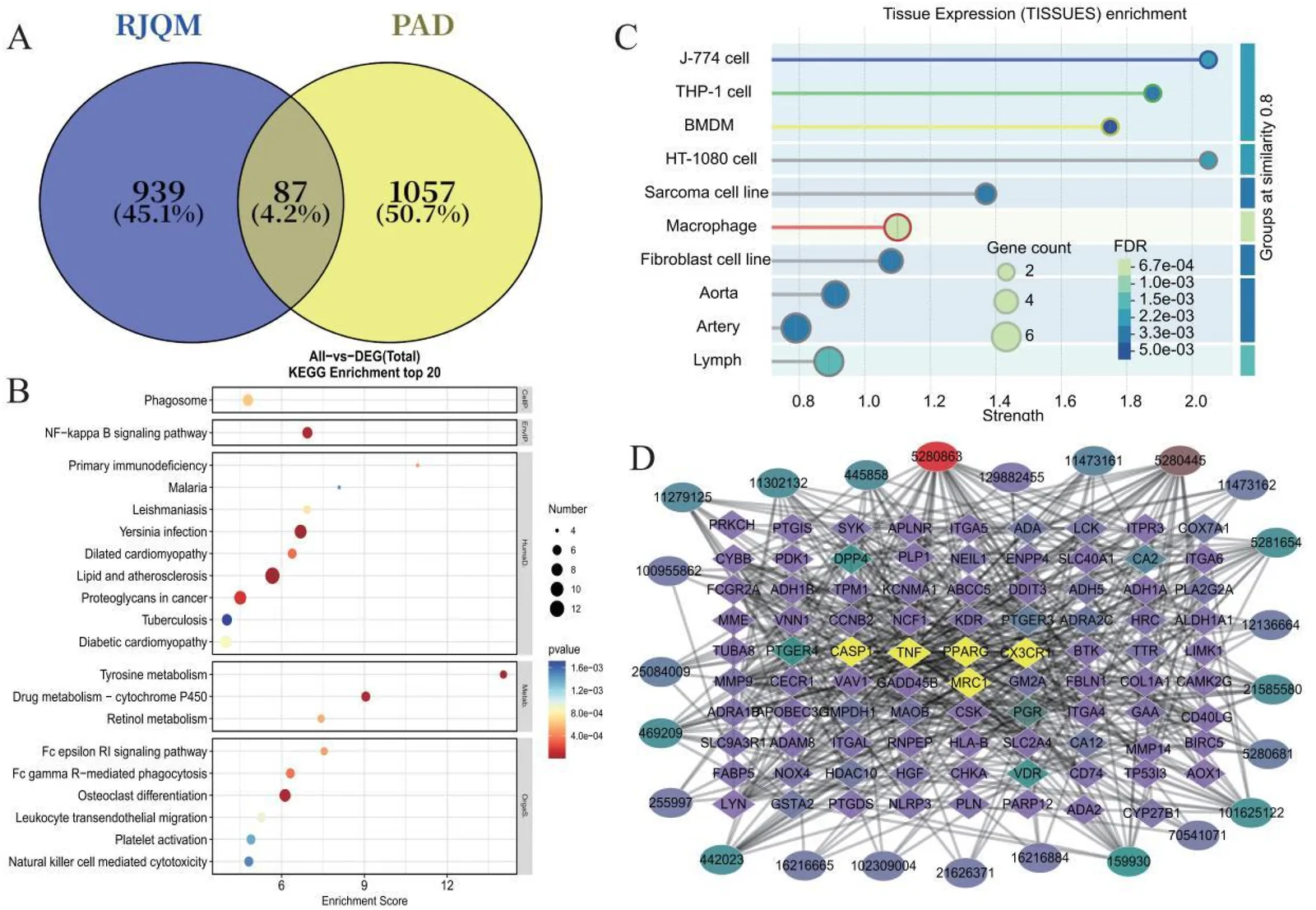
Identification of potential targets and key active components of RJQM against PAD. **(A)** Venn analysis of targets between RJQM and PAD. **(B)** KEGG pathway enrichment analysis of 87 overlapping targets between RJQM and PAD. **(C)** Tissue expression enrichment analysis indicating predominant localization of the shared targets in macrophages. **(D)** Compound–target interaction network showing kaempferol as the core compound with the highest degree value.

A total of 1,026 potential targets of RJQM absorbed components were retrieved from multiple databases and intersected with DEGs identified in PAD, yielding 87 overlapping targets (**Figure 3A**). KEGG pathway enrichment analysis revealed that these genes were significantly involved in immune and inflammatory signaling pathways, including NF-κB signaling, leukocyte transendothelial migration, Fc gamma R-mediated phagocytosis, and lipid and atherosclerosis pathways (**Figure 3B**). Tissue expression enrichment analysis further demonstrated that the shared targets were predominantly expressed in macrophages, including THP-1 cells, BMDMs, and J-774 cells, suggesting that RJQM may exert therapeutic effects on PAD through the modulation of macrophage-mediated inflammation (**Figure 3C**). Network construction of component–target interactions revealed that kaempferol (PubChem CID: 5280863) exhibited the highest degree value within the network, implicating it as a potential key bioactive compound responsible for the therapeutic effects of RJQM on PAD (**Figure 3D**).

### Kaempferol target acquisition from RNA sequencing and public database

**Figure 4:**
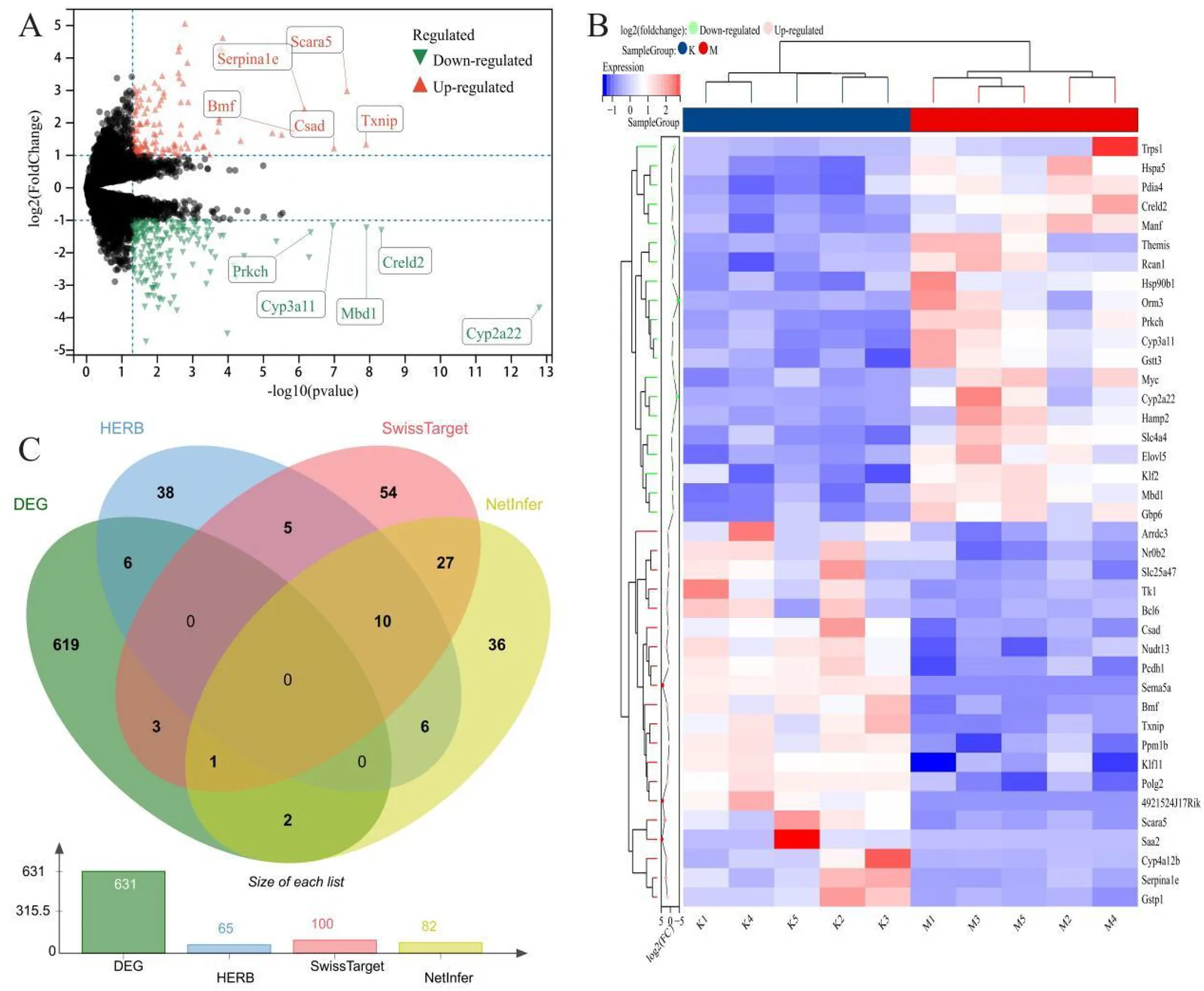
Kaempferol target acquisition from RNA sequencing and public database. **(A)** Volcano plot depicting the distribution of differentially expressed genes. **(B)** Heatmap showing the gene expression profiles across samples, grouped into two categories (Kaempferol and Model). **(C)** Venn diagram illustrating the overlap of Kaempferol DEGs with predicted drug targets from three databases: HERB, SwissTarget, and NetInfer.

To define the molecular targets of kaempferol, we analyzed the GSE145665 dataset, comparing gene expression in high-fat diet mice treated with kaempferol versus untreated controls. This identified 631 differentially expressed genes (|fold change| ≥ 1.5 and FDR value < 0.05), including 254 up-regulated genes and 377 down-regulated genes (**Figure 4A**). In the top 30 genes presented in the heatmap analysis, up-regulated genes include Trps1, Hspa5, Pdia4, Creld2, Manf, etc., and down-regulated genes include Arrdc3, Nr0b2, Slc25a47, Tk1, Bcl6, etc. (**Figure 4B**). To comprehensively map kaempferol’s potential interactome, we integrated these DEGs with predicted targets from the HERB, SwissTargetPrediction, and NetInfer databases. After merging, we compiled a robust set of 807 unique kaempferol-associated targets for downstream analysis (**Figure 4C**). This integrated approach provides a broad foundation for investigating kaempferol’s mechanism of action.

### Kaempferol’s potential therapeutic effects on PAD: target, enrichment function, and macrophage-associated molecular mechanisms

**Figure 5:**
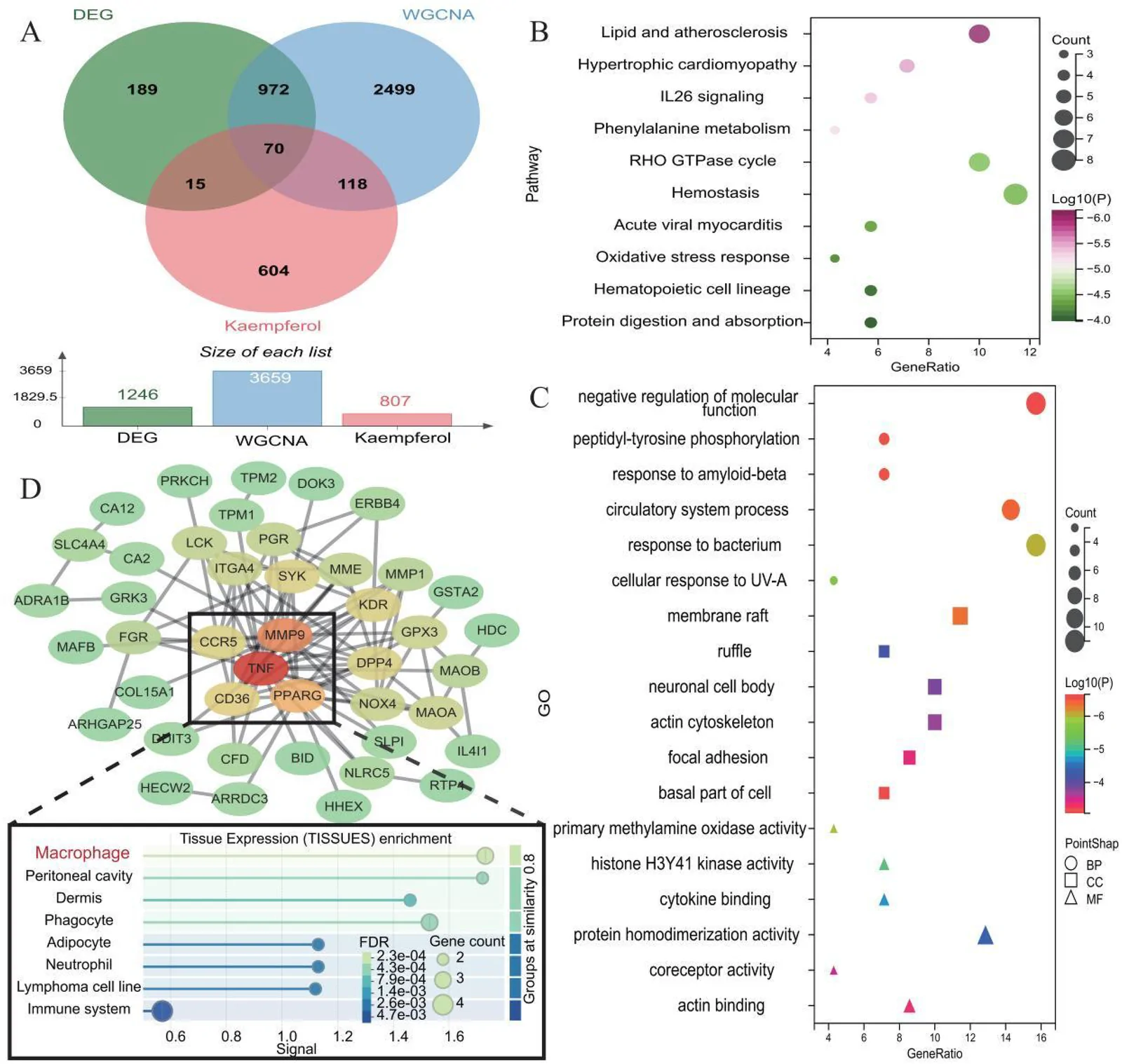
Kaempferol’s therapeutic effects on PAD: target, function, and macrophage mechanisms. **(A)** Venn diagram and bar chart illustrating the overlap and size of DEG, WGCNA, and Kaempferol-related gene sets. **(B)** Bubble plot depicting the pathway analysis results for the overlap genes. **(C)** Bubble plot showing the GO analysis results for the overlap genes. Biological process (BP), cellular component (CC), and molecular function (MF). **(D)** Protein-protein interaction network and tissue expression enrichment analysis.

To pinpoint the specific targets through which kaempferol might exert therapeutic effects in PAD, we intersected the PAD-associated genes (DEGs from GSE100927 and key genes from WGCNA modules) with the compiled kaempferol targets. This integration revealed 70 overlapping targets (**Figure 5A**), representing prime candidates mediating kaempferol’s action against PAD. Pathway analysis (**Figure 5B**) highlights significant pathways such as lipid and atherosclerosis, hypertrophic cardiomyopathy, and IL26 signaling. Gene ontology (GO) analysis (**Figure 5C**) elucidates various biological processes, molecular functions, and cellular components associated with Kaempferol, including negative regulation of molecular function, peptidyl-tyrosine phosphorylation, membrane raft, ruffle, primary methylamine oxidase activity and histone H3Y41 kinase activity. Protein-protein interaction (PPI) network analysis identified TNF, MMP9, PPARG, CD36, and CCR5 as highly connected hubs within this target set. Crucially, tissue expression enrichment analysis indicated that ‘macrophage’ was the most significantly enriched term for these 70 kaempferol-PAD targets (**Figure 5D**), strongly suggesting macrophages as a primary cellular site of kaempferol’s therapeutic activity in PAD.

### Integrated infiltrating immune profiling and macrophage-centric mechanistic insights reveal kaempferol’s therapeutic targets in PAD pathogenesis

**Figure 6:**
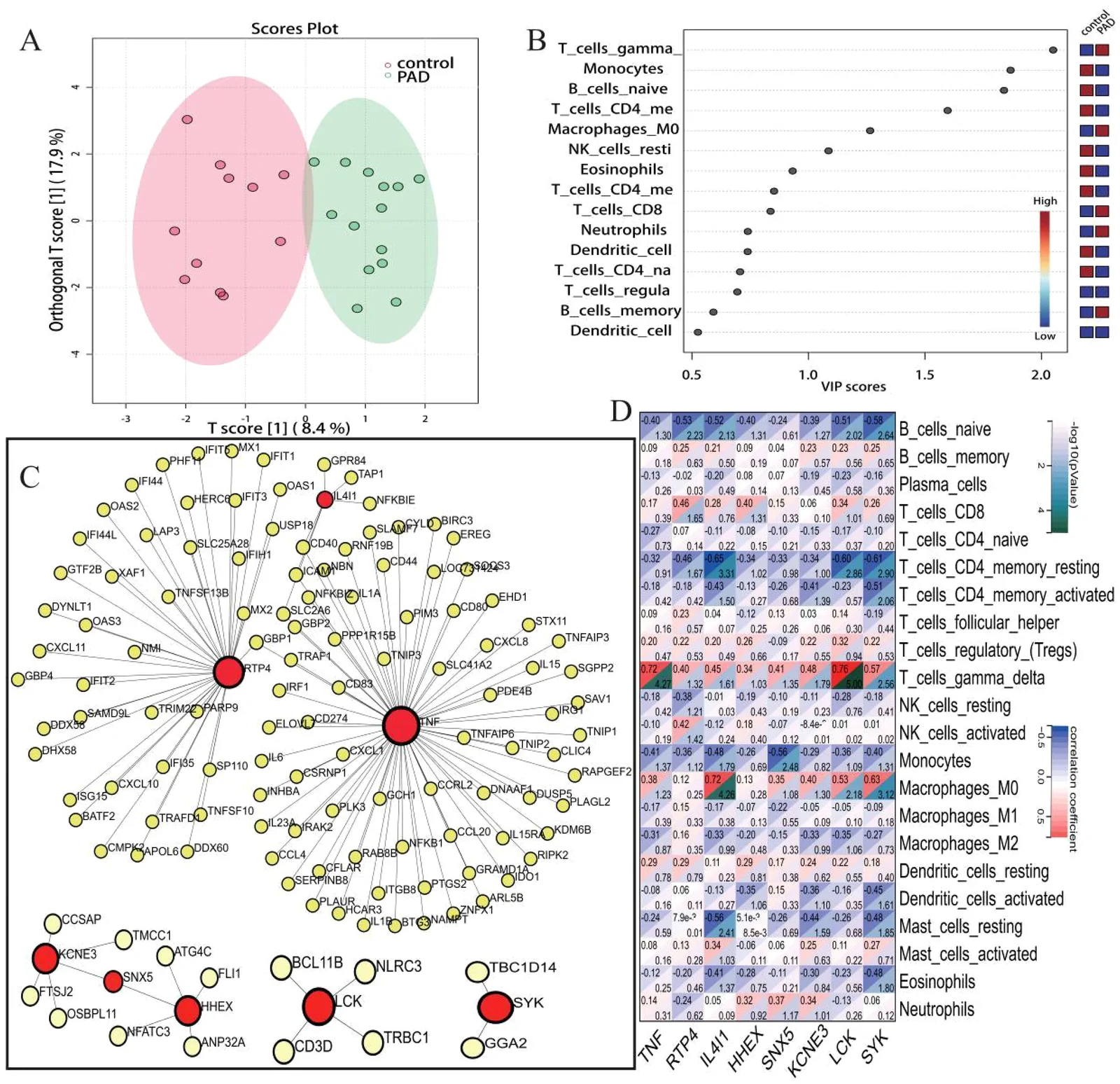
Infiltrating immune profiling and macrophage insights reveal kaempferol’s targets in PAD. **(A)** The scores plot from the OPLS-DA shows the separation between control and PAD samples based on their infiltrating immune profiling profiles. **(B)** This panel displays the VIP scores for various immune cell types. **(C)** The network plot visualizes the interactions between key genes involved in the macrophage response. Red nodes represent key genes in macrophage, while yellow nodes represent other related genes. **(D)** The heatmap shows the correlation between different immune cell types and gene expression levels.

Analysis of immune cell infiltration using CIBERSORTx revealed a significantly altered immune landscape in PAD arteries compared to controls, effectively separable by OPLS-DA (**Figure 6A**). Key infiltrating cell types distinguishing PAD included T_cells_gamma_delta, Monocytes, and Macrophages_M0 (**Figure 6B**). Building on the macrophage enrichment of kaempferol-PAD targets, we performed cell type-specific enrichment analysis. This identified eight kaempferol-PAD targets (TNF, RTP4, IL4I1, HHEX, SNX5, KCNE3, LCK, SYK) with significant enrichment specifically in macrophages (**Figure 6C**). Furthermore, correlation analysis demonstrated that several of these macrophage-enriched targets (e.g., TNF, LCK, SYK, IL4I1, KCNE3) also showed significant associations with gamma delta T cell infiltration (**Figure 6D**), suggesting potential crosstalk or shared regulatory mechanisms within the PAD immune microenvironment. These findings solidify macrophages as central cellular mediators of kaempferol’s pharmacological effects in PAD.

### Single-cell transcriptomic profiling reveals cellular heterogeneity and dysregulation in skeletal muscle of PAD

**Figure 7:**
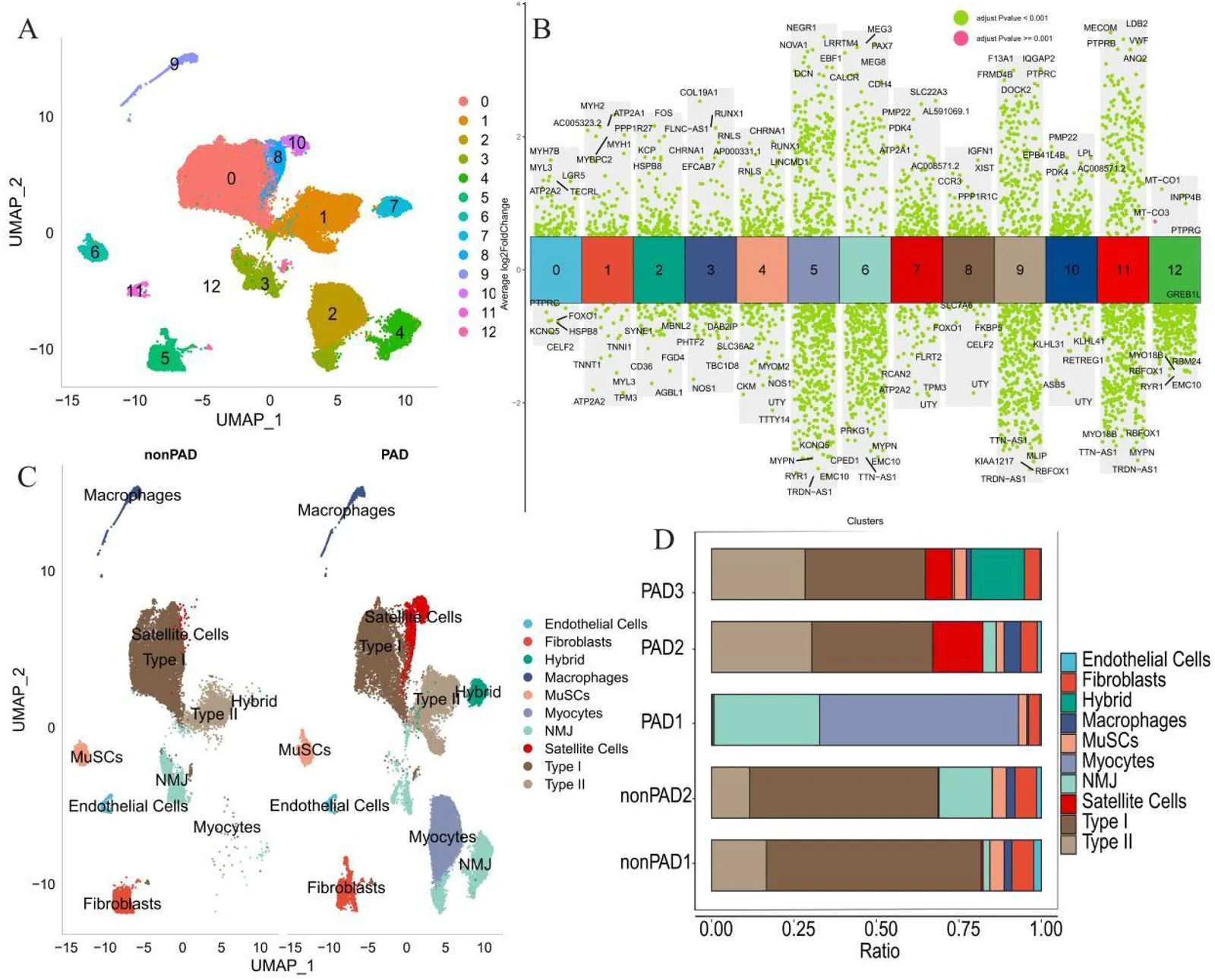
Single-cell sequencing unveils cellular composition and PAD-associated heterogeneity. **(A)** UMAP visualization of 13 cell clusters (0–12) identified via single-cell RNA sequencing. **(B)** Marker gene expression profiles for each cell cluster, with genes specifically expressed in corresponding clusters highlighted. **(C)** Cellular composition comparison between PAD and non-PAD groups, displaying distributions of cell types. **(D)** Proportional representation of cell types (Endothelial Cells, Fibroblasts, Macrophages, etc.) in PAD-related (PAD1–3) and non-PAD (nonPAD1–2) clusters, illustrating compositional heterogeneity.

To dissect cellular heterogeneity within the PAD microenvironment, we analyzed scRNA-seq data (GSE233882) from skeletal muscle of PAD patients and controls. Unsupervised clustering identified 13 distinct cellular clusters (**Figure 7A**). To identify the molecular signatures of each cluster, we performed differential gene expression analysis. The heatmap in **Figure 7B** shows the average log2 fold change of DEGs across the 13 clusters, including 0 (MYH7B, MYL3), 1 (MYH2, TP2A1), 2 (FOS, FLNC-AS1), 3 (COL19A1, RUNX1), 4 (CHRNA1, RUNX1), 5 (NEGR1, LRRTM4), 6 (MEG3, PAX7), 7 (SLC22A3, PMP22), 8 (IGFN1, XIST), 9 (F13A1, QGAP2), 10 (PMP22, LPL), 11 (LDB2, MECOM), 12 (MT-CO1, INPP4B). The UMAP projection in **Figure 7C** illustrates the distribution of annotated cell types, including endothelial cells, fibroblasts, Type I (slow muscle fiber), Type II (fast muscle fiber), hybrid cells (Type I and Type II), macrophages, MuSCs (muscle stem cells), myocytes, NMJ (neuromuscular junction associated muscle fibers), fibroblasts, and satellite cells. Comparative quantification revealed a significant shift in cellular composition in PAD tissue (**Figure 7D**). Notably, while macrophage proportions remained relatively uniform across nonPAD samples, their infiltration in PAD samples exhibited profound inter-sample heterogeneity, with specific ischemic niches (e.g., PAD2) demonstrating massive macrophage expansion. This altered, highly heterogeneous cellular landscape underscores the complex, spatially dynamic involvement of immune cell populations in PAD skeletal muscle pathology.

### Expression analysis of kaempferol targets in PAD skeletal muscle single-cell transcriptomics

**Figure 8:**
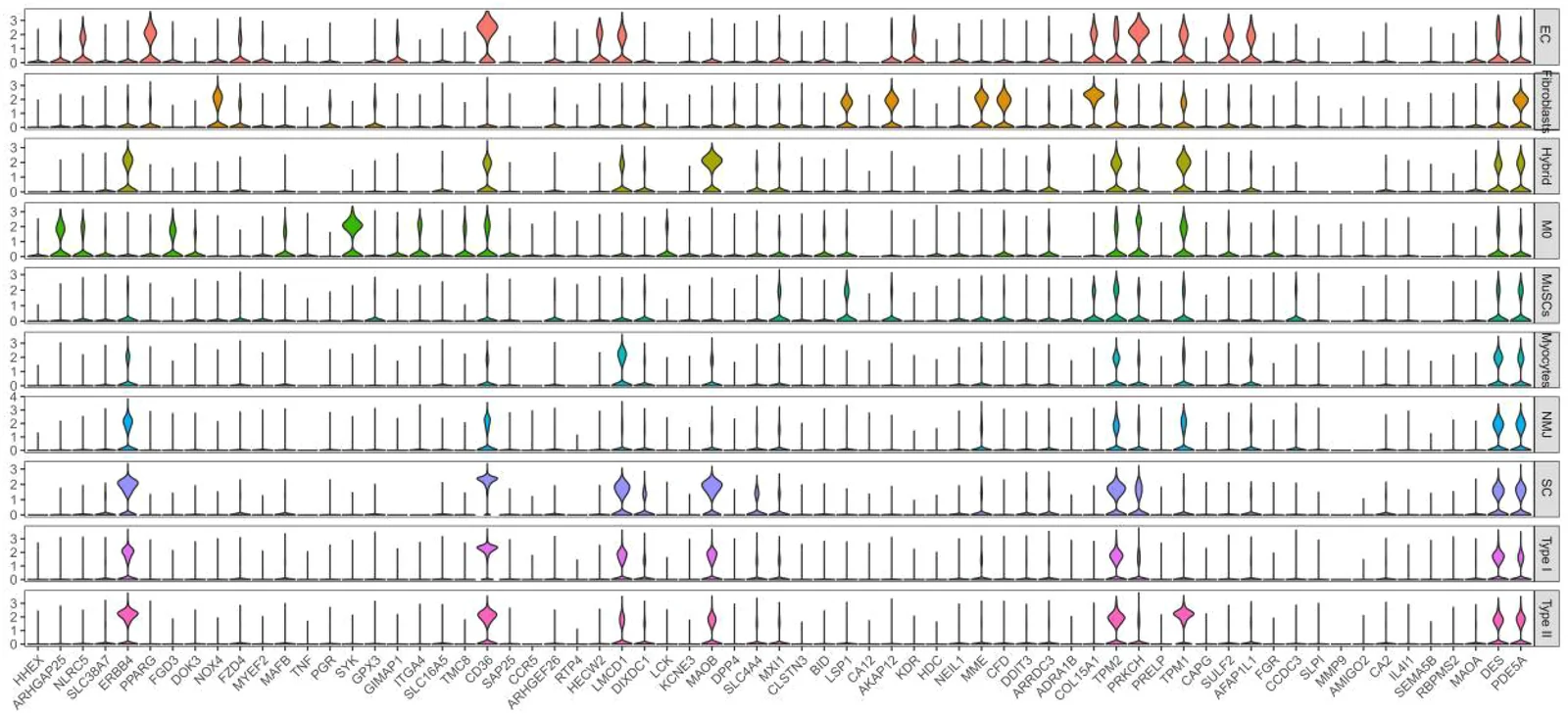
Expression profiles of kaempferol targets in PAD skeletal muscle single-cell transcriptomics. Violin plots showing the expression levels of 70 kaempferol targets across various cell types identified in the skeletal muscle microenvironment.

We next mapped the expression patterns of the 70 kaempferol-PAD targets within this resolved skeletal muscle cellular atlas. Expression analysis demonstrated that many key targets, including CD36, PRKCH, SYK, and NLRC5, were predominantly expressed in macrophages (MO) and endothelial cells (EC) within the PAD microenvironment (**Figure 8**). COL15A1 and PDE5A showed fibroblast (FB) enrichment, while MAOB and TPM1 were elevated in hybrid cells. This cell-type-specific expression profiling corroborates the macrophage and endothelial enrichment identified in bulk tissue analysis and highlights these cell types, particularly macrophages, as critical sites where kaempferol’s targets are active in the PAD tissue context.

### hdWGCNA analysis identifies gene modules linked to macrophage polarization in PAD skeletal muscle

**Figure 9:**
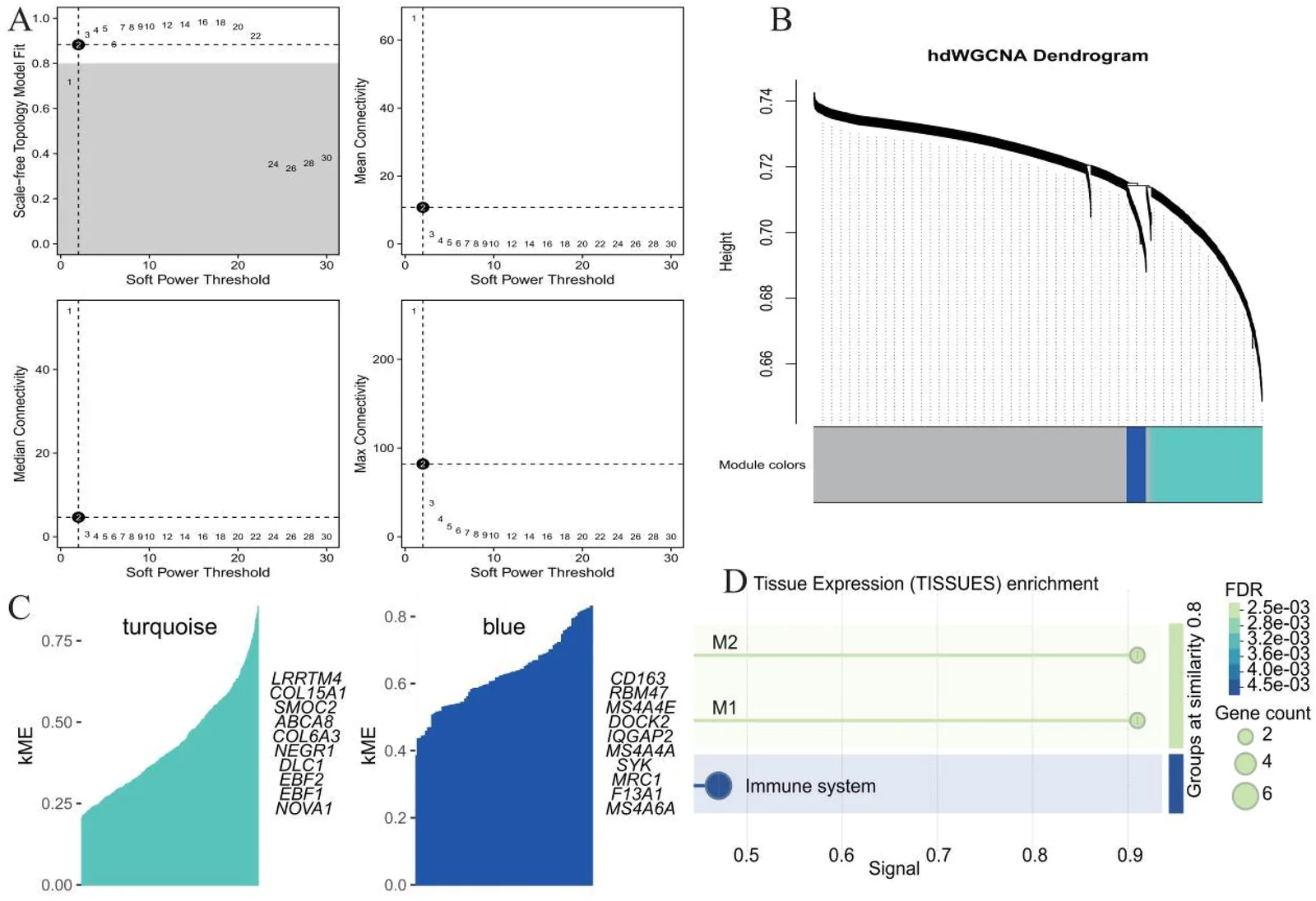
hdWGCNA analysis of PAD skeletal muscle single-cell sequencing data. **(A)** Selection of the optimal soft power threshold for constructing a scale-free network. The scale-free topology model fit, mean connectivity, median connectivity, and maximum connectivity are plotted against various soft power thresholds. **(B)** hdWGCNA dendrogram showing the hierarchical clustering of genes into distinct modules. The blue and turquoise modules are highlighted. **(C)** Module eigengene (kME) distribution for the turquoise and blue modules. The top 10 genes in each module are listed. **(D)** Tissue expression enrichment analysis using the TISSUES database. The blue module is significantly enriched in the immune system, particularly in macrophage polarization (M2 and M1), while the turquoise module does not show significant enrichment.

To identify gene co-expression networks specifically associated with PAD at the single-cell level, particularly within macrophage populations, we performed hierarchical directed WGCNA (hdWGCNA) on the skeletal muscle scRNA-seq data. This analysis identified key gene modules, notably the blue and turquoise modules (**Figures 9A, B**). Tissue expression enrichment analysis revealed that genes within the blue module were significantly enriched for terms related to macrophage polarization (both M1 and M2 states) and immune system processes (**Figures 9C, D**). In contrast, the turquoise module showed no significant enrichment in specific immune processes. The blue module contained key macrophage-related genes like CD163, SYK, MRC1, and F13A1, reinforcing the link between macrophage gene networks and PAD pathophysiology in skeletal muscle. Within the hdWGCNA framework, robust macrophage polarization modules comprising hundreds of highly co-expressed genes were identified. The indicated gene count of 2 does not reflect the total size of these biological modules; rather, it highlights the highly specific pharmacological intersection between these massive networks and the 70 verified kaempferol targets. Because these specific overlapping targets function as high-centrality master hub genes, their modulation indicates a highly targeted pharmacological intervention rather than an isolated statistical artifact.

### Kaempferol attenuates LPS-induced inflammation and M1 polarization

**Figure 10:**
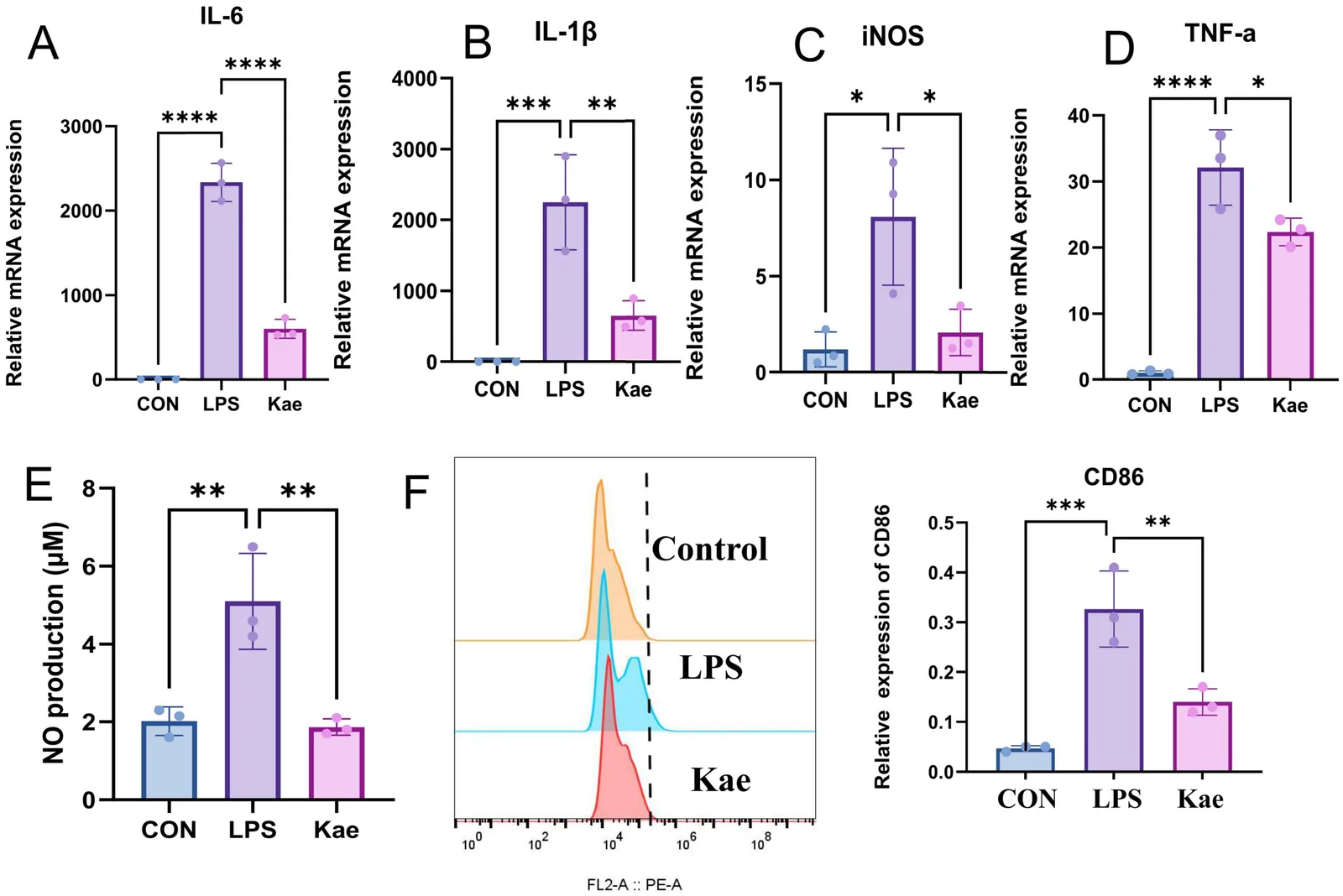
Kaempferol reduces LPS-induced inflammation and M1 polarization. **(A)** IL-6, **(B)** IL-1β, **(C)** iNOS, and **(D)** TNF-α mRNA expression levels in macrophages treated with CON, LPS, or LPS + Kaempferol. **(E)** NO production measured in cell supernatants. **(F)** Flow cytometric analysis of CD86 surface expression to evaluate M1 macrophage polarization. Data are presented as mean ± SEM (n = 3). Statistical significance was determined using one-way ANOVA with Tukey’s *post-hoc* test: *p < 0.05, **p < 0.01, ***P < 0.001, ****p < 0.0001, ns: not significant.

To functionally validate the bioinformatically predicted anti-inflammatory and macrophage-modulating effects of kaempferol, we performed *in vitro* experiments using LPS-stimulated RAW264.7 macrophages. LPS treatment robustly induced a pro-inflammatory M1-like state, characterized by significantly increased mRNA expression of IL-6, IL-1β, iNOS, and TNF-α, elevated NO production, and enhanced surface expression of the M1 macrophage marker CD86 measured by flow cytometry. Critically, treatment with kaempferol significantly attenuated the LPS-induced upregulation of all these inflammatory markers and reduced NO production, and markedly lowered CD86 positivity in LPS-challenged macrophages (**Figure 10**). These results demonstrate that kaempferol effectively suppresses LPS-triggered inflammation and inhibits M1 polarization in macrophages, providing experimental support for its predicted mechanism of action derived from multi-omics integration.

### Ferroptosis driver scores and kaempferol-macrophage-targeted gene correlations in PAD

**Figure 11:**
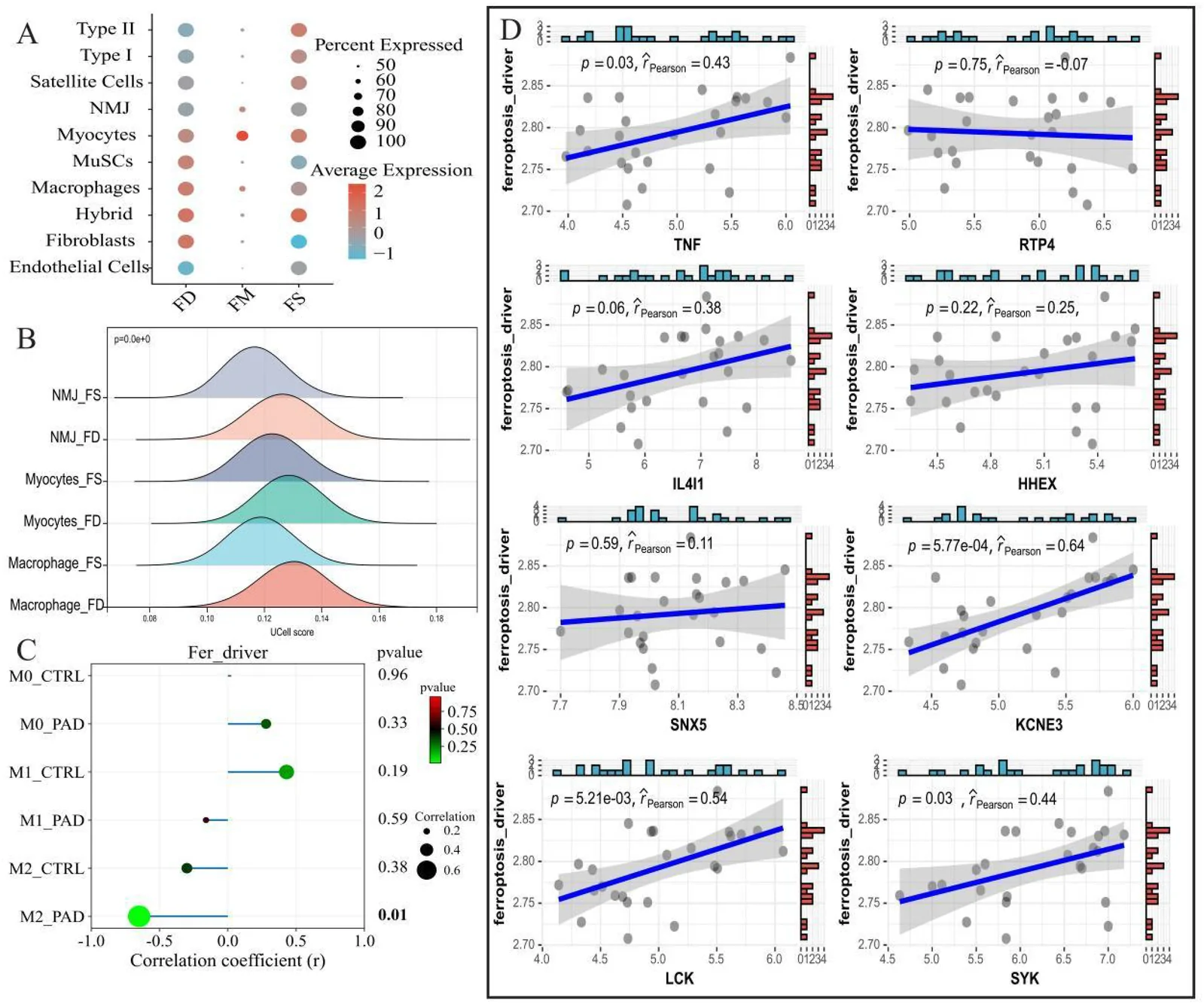
Ferroptosis driver scores and macrophage-targeted gene correlations in PAD. **(A)** Ferroptosis scores for various cell types, with myocytes, macrophages, and NMJ exhibiting the highest scores under the FM condition. **(B)** UCell scores for different cell types and conditions (FD, FM, FS), with macrophages showing the highest ferroptosis driver scores. **(C)** Correlation coefficients and p-values for ferroptosis driver genes under different conditions, highlighting significant changes in PAD conditions. **(D)** Scatter plots and Pearson correlation coefficients for the relationship between ferroptosis driver scores and various genes (TNF, RTP4, IL4I1, HHEX, SNX5, KCNE3, LCK, SYK) targeted by kaempferol in macrophages, with significant correlations indicated. FM, Ferroptosis marker. FS, Ferroptosis suppressor. FD, Ferroptosis driver.

Given the emerging role of ferroptosis in vascular pathologies and its potential link to macrophage function, we investigated ferroptosis activity across cell types in the PAD skeletal muscle microenvironment using the scRNA-seq data. Myocytes, macrophages (MO), and NMJ-associated fibers exhibited the highest ferroptosis marker scores (**Figure 11A**). Importantly, macrophages displayed significantly elevated ferroptosis driver scores compared to suppressor scores, indicating a propensity for ferroptosis susceptibility in these cells within PAD (**Figure 11B**). Analysis of vascular ferroptosis scores in relation to macrophage infiltration revealed a significant negative correlation specifically between vascular ferroptosis scores and M2 macrophage infiltration scores under PAD conditions, a relationship absent in controls (**Figure 11C**). Furthermore, within macrophages themselves, ferroptosis driver scores showed significant positive correlations with the expression levels of several key kaempferol targets, including TNF, SNX5, LCK, and SYK (**Figure 11D**). This suggests that kaempferol’s modulation of these macrophage targets may be functionally linked to ferroptosis regulation in the context of PAD.

We next performed virtual knockout of TNF, SNX5, LCK and SYK across all macrophage populations and conducted GO enrichment analysis on differentially regulated genes to dissect their transcriptional regulatory roles. Virtual knockout of TNF and LCK only yielded pathways associated with striated muscle contraction, which are regarded as technical artifacts derived from contaminating muscle nuclear transcripts in snRNA-seq data, consistent with their extremely low or absent expression in macrophages. In contrast, virtual deletion of SNX5 produced abundant differentially regulated genes prominently enriched in immune receptor and B cell activation signaling cascades. SYK knockout also enriched B cell activation-related immune pathways, though the number of altered genes was limited, leading to relatively weak enrichment power (**Supplementary Figure 1**). Collectively, in silico perturbation data demonstrate that only SNX5 and SYK exert authentic immune regulatory functions in human PAD macrophages, while TNF and LCK lack macrophage-specific regulatory effects.

### Inflammatory stratification of PAD macrophages reveals cellular targets for kaempferol

**Figure 12:**
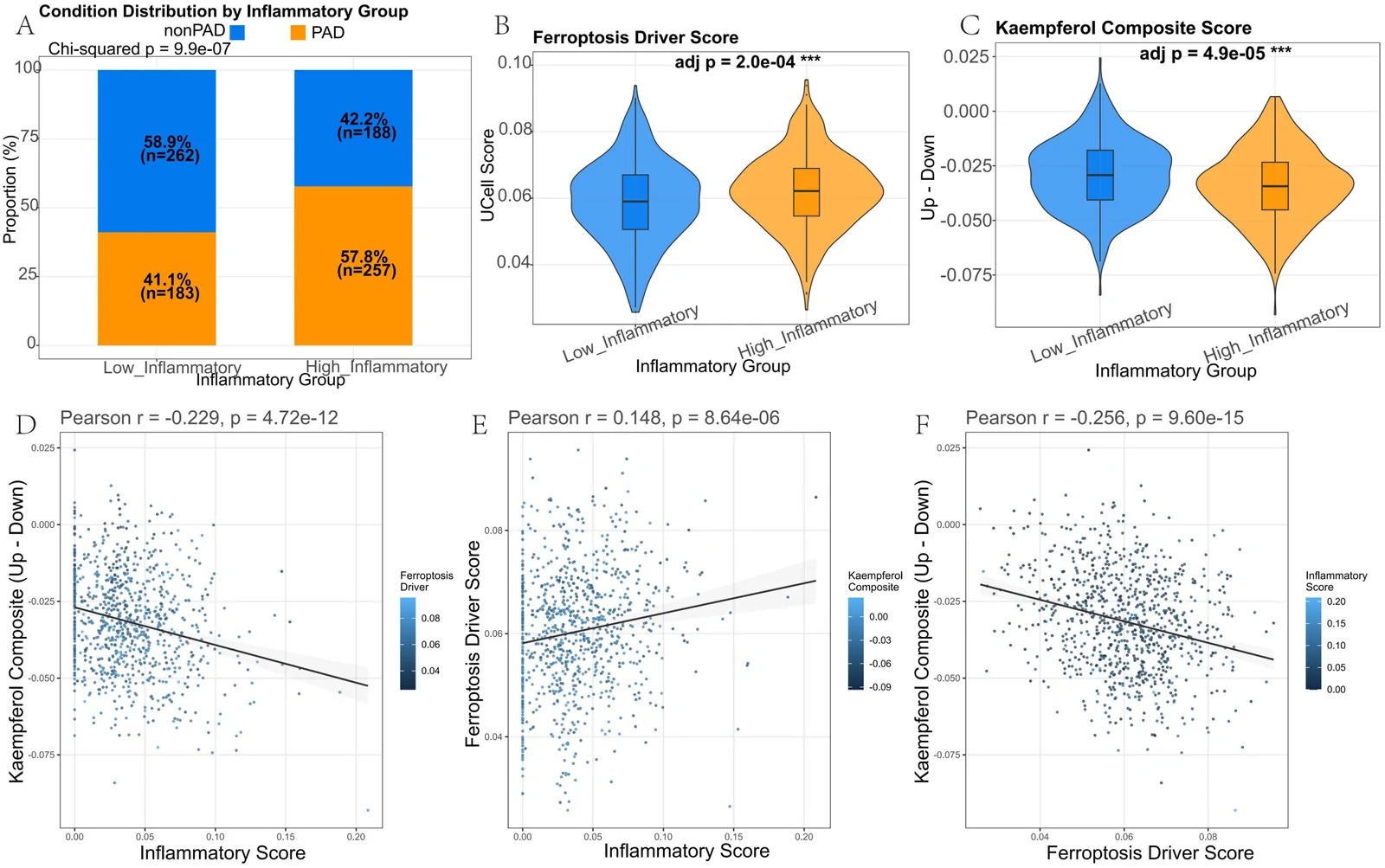
Transcriptional profiling of inflammatory stratified macrophages from human PAD samples. **(A)** Bar chart illustrating the proportion of non-PAD and PAD macrophages distributed within low-inflammatory and high-inflammatory subgroups. Chi-squared test was used to assess group distribution differences. **(B)** Violin plot comparing ferroptosis driver signature scores between low-inflammatory and high-inflammatory macrophage subsets. **(C)** Violin plot of kaempferol composite scores (upregulated signature minus downregulated signature) across inflammatory subgroups. **(D)** Pearson correlation between inflammatory score and kaempferol composite score in macrophages. **(E)** Pearson correlation between inflammatory score and ferroptosis driver score in macrophages. **(F)** Pearson correlation between ferroptosis driver score and kaempferol composite score in macrophages. Adjusted p-values are shown for violin plot comparisons. ***P < 0.001.

Condition distribution analysis revealed robust enrichment of PAD-derived macrophages within the high-inflammatory subgroup (57.8% PAD macrophages, compared with 41.1% in the low-inflammatory group), confirming that the ischemic microenvironment of PAD drives macrophage inflammatory activation (**Figure 12A**). We next compared ferroptosis driver signature scores between the two macrophage subgroups. High-inflammatory macrophages exhibited significantly elevated ferroptosis driver scores (**Figure 12B**), consistent with a positive Pearson correlation between inflammatory score and ferroptosis driver score across all macrophages (**Figure 12E**). These data demonstrate that macrophages with heightened inflammatory status simultaneously display enhanced ferroptosis transcriptional signatures in PAD lesions.We further calculated a kaempferol composite score (upregulated signature subtracted from downregulated signature) to evaluate cell-subset-specific drug responsiveness. The kaempferol composite score was markedly reduced in high-inflammatory macrophages (**Figure 12C**). Correlation analysis showed the kaempferol composite score negatively correlated with inflammatory score (Figur12D) and ferroptosis driver score (**Figure 12F**). Collectively, these results indicate that transcriptional programs suppressed by kaempferol are preferentially activated within high-inflammatory macrophages.

### Molecular docking validation of kaempferol with eight macrophage-related targets

**Figure 13:**
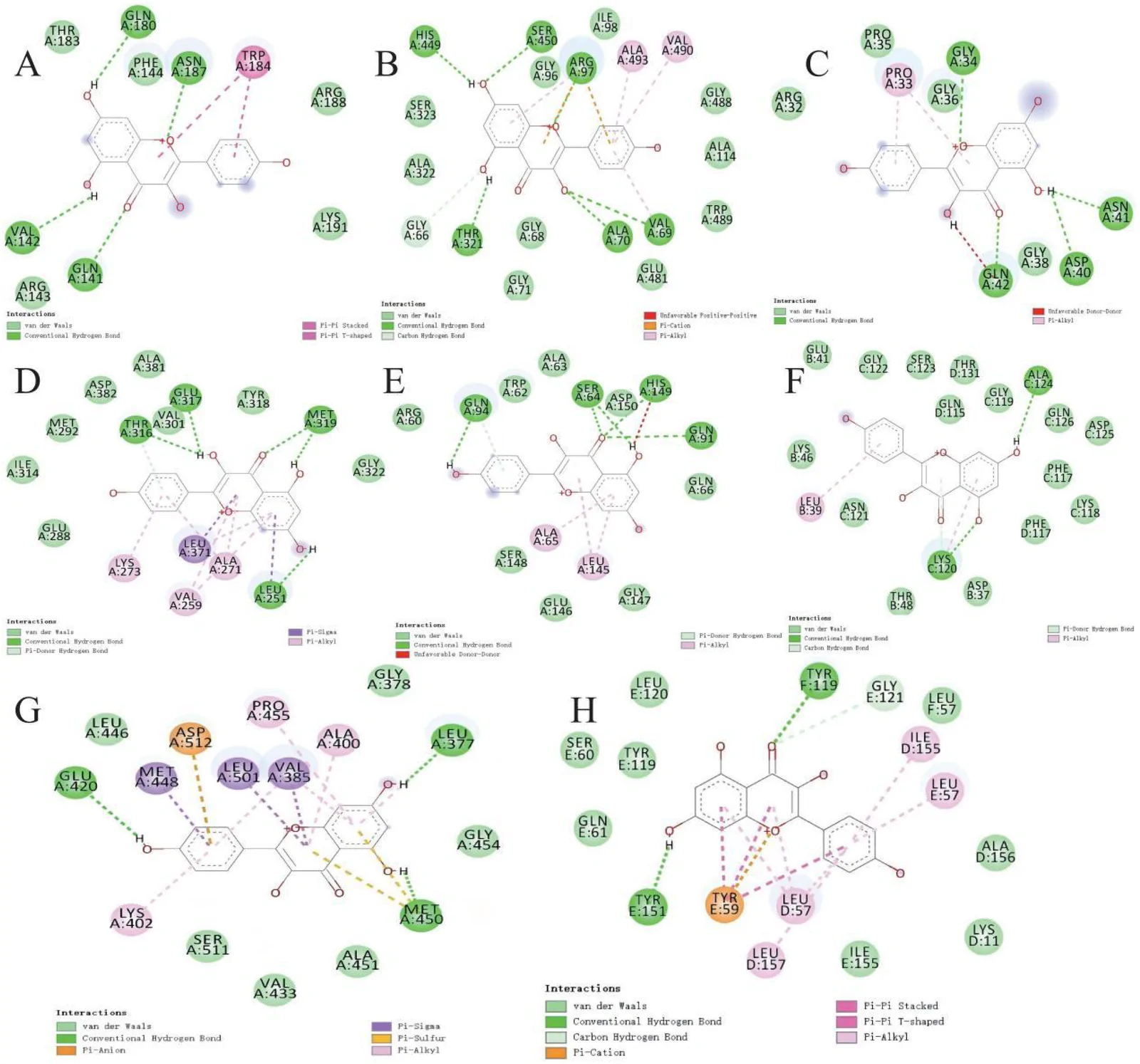
Molecular docking of kaempferol with eight macrophage-related targets. The 2D interaction diagrams display the binding modes of kaempferol with **(A)** HHEX, **(B)** IL4I1, **(C)** KCNE3, **(D)** LCK, **(E)** RTP4, **(F)** SNX5, **(G)** SYK, and **(H)** TNF.

To assess the structural feasibility of kaempferol directly interacting with the key macrophage-enriched targets, we performed molecular docking simulations. Kaempferol exhibited robust predicted binding affinities across the majority of identified targets. Notably, IL4I1 displayed the strongest affinity (ΔG = -9.2 kcal/mol), followed by TNF (ΔG = -8.9 kcal/mol), LCK (ΔG = -8.4 kcal/mol), SYK (ΔG = -7.9 kcal/mol), RTP4 (ΔG = -7.8 kcal/mol), and SNX5 (ΔG = -7.7 kcal/mol). HHEX and KCNE3 showed moderate to lower affinities of -6.4 kcal/mol and -5.4 kcal/mol, respectively (**Supplementary Table 2**). To validate the docking protocol, we compared the binding energies of kaempferol with the native ligands (positive controls) co-crystallized within the TNF, SYK, and LCK structures. The binding affinities of kaempferol were comparable to those of the native ligands (LCK: -8.4 vs. -9.5 kcal/mol; SYK: -7.9 vs. -8.5 kcal/mol), suggesting that kaempferol occupies the active pockets with high stability. Visualization of the binding poses (**Figure 12**) revealed that kaempferol establishes critical hydrogen bonds and Pi-cation/Pi-Pi interactions with key amino acid residues, such as LEU251 in HHEX (**Figure 13A**), HIS449 in IL4I1 (**Figure 13B**), GLY34 inKCNE3 (**Figure 13C**), MET319 in LCK (**Figure 13D**), GLN91 in RTP4 (**Figure 13E**), ALA124 in SNX5 (**Figure 13F**), LEU377 in SYK (**Figure 13G**), and TYR119 in TNF (**Figure 13H**). These results provide structural support for kaempferol’s potential to directly engage and modulate these critical macrophage targets implicated in PAD pathogenesis.

### Kaempferol rescues impaired angiogenesis in zebrafish vascular injury model to support its protective role in PAD

**Figure 14:**
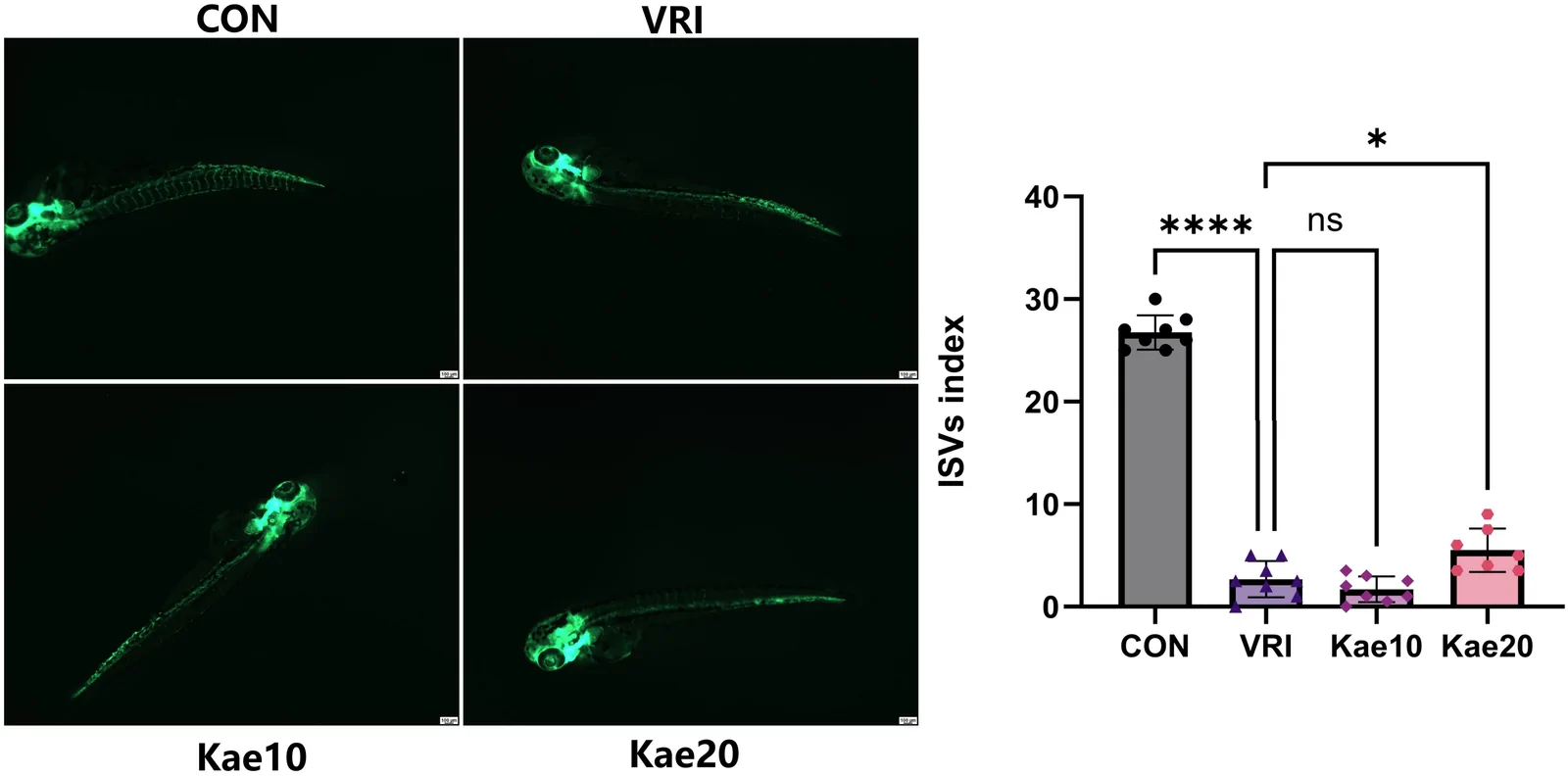
Representative fluorescence images of intersegmental vessels in zebrafish larvae 48 h post-injury. Vessels are labeled in green. *P < 0.05, ****P < 0.0001, ns: not significant.

To validate the translational functional outcome predicted by our human single-cell multi-omics analysis, we constructed a zebrafish vascular regeneration injury model to characterize kaempferol’s *in vivo* angiogenic activity under ischemic vascular damage. Consistent with our omics-derived hypothesis that kaempferol remodels the disordered inflammatory-iron metabolic microenvironment to facilitate vascular repair, quantitative intersegmental vessel (ISV) scoring revealed severe vascular destruction and reduced ISV density in the VRI injury group. Low-dose kaempferol treatment produced non-significant improvement of vessel integrity. In contrast, high-dose kaempferol markedly restored damaged intersegmental vasculature and elevated the ISV composite score, demonstrating robust pro-angiogenic capacity upon vascular injury (**Figure 14**). Collectively, this vertebrate *in vivo* evidence functionally corroborates our single-cell mechanistic findings in human PAD macrophages, further confirming that kaempferol exerts protective effects against ischemic vascular lesions.

## Discussion

Peripheral artery disease (PAD) is a prevalent and often underdiagnosed condition characterized by the narrowing and blockage of peripheral arteries, primarily due to atherosclerosis. This disease significantly impacts the quality of life, leading to symptoms such as intermittent claudication, pain, and, in severe cases, critical limb ischemia (22). Patients with PAD not only experience functional limitations but also face increased risks of cardiovascular morbidity and mortality, including myocardial infarction and stroke (23). The prevalence of PAD rises with age, affecting approximately 10% of individuals over the age of 60 and even higher rates among those with diabetes and hypertension (24). Given the substantial burden of this disease, effective therapeutic strategies and a deeper understanding of its underlying mechanisms are imperative.

While existing studies have documented the general cardiovascular protective effects of kaempferol, the precise molecular mechanisms within the complex PAD microenvironment have remained unclear. Our core innovation lies in a dual-track regulatory model built on single-cell inflammatory stratification and in silico gene knockout, which decouples macrophage inflammatory signaling from ferroptosis. The integrated multi-omics data strongly supports the hypothesis that rather than merely acting as a general anti-inflammatory agent, kaempferol’s therapeutic efficacy is intimately associated with its ability to concurrently modulate the coupled inflammatory-ferroptotic microenvironment. This positions the macrophage-ferroptosis axis as a highly probable dual-action therapeutic target in the progression of PAD that warrants rigorous future in vivo investigation (25). This research not only contributes to the understanding of PAD’s pathophysiology but also highlights the potential for kaempferol as a multi-target therapeutic agent, paving the way for future studies and clinical applications.

By reconstructing the transcriptional landscape of the disease, our study identified 1,144 DEGs in PAD, reflecting a profound dysregulation of vascular and immune homeostasis. This was characterized by the significant up-regulation of genes such as HMGA1P4, MARCKS, and MYOC, and down-regulation of PLAA, PPP1R9A, and CHODL. The overexpression of HMGA1P4, a lncRNA linked to chemoresistance in cancers, may drive vascular smooth muscle cell (VSMC) proliferation in PAD by modulating the miR-301b/CCNA2 axis, as shown in gastric cancer studies (26, 27). Similarly, MARCKS, a regulator of cytoskeletal dynamics and VSMC proliferation, could promote intimal hyperplasia through its role in phosphatidylinositol 4,5-bisphosphate metabolism (28, 29). The unexpected up-regulation of MYOC, traditionally associated with glaucoma, suggests a novel vascular role via TGF-β signaling and ER stress pathways (30). Conversely, suppressed PLAA and CHODL may impair angiogenesis, exacerbating endothelial dysfunction.

The present study also delineates kaempferol’s therapeutic mechanisms in PAD through multi-omics integration, revealing its pleiotropic effects on lipid metabolism, macrophage polarization, and inflammatory pathways (31). RNA sequencing identified 631 DEGs in kaempferol-treated mice, with key up-regulated targets such as Hspa5, involved in regulating endoplasmic reticulum stress (32), and down-regulated genes including Nr0b2, a modulator of gluconeogenesis (33), both linked to metabolic regulation and oxidative stress mitigation. Rather than evaluating these targets in isolation, our intersection analysis synthesized bulk datasets to map these targets directly to the disease pathology. This pinpointed 70 overlapping targets between kaempferol and PAD pathogenesis, with hub nodes TNF (34), MMP9 (35), PPARG (36), and CD36 (37) collectively modulating atherosclerosis progression via endothelial activation, lipid uptake, and foam cell formation. Tissue enrichment analysis highlighted macrophages as central mediators (38), supported by kaempferol’s suppression of pro-inflammatory IL-26 signaling and enhancement of M2 polarization pathways such as peptidyl-tyrosine phosphorylation, critical for plaque stabilization (39). The interaction between kaempferol and CCR5—a chemokine receptor linked to monocyte recruitment —further suggests its capacity to attenuate immune cell infiltration, a hallmark of advanced PAD lesions (40). Furthermore, pathway analysis underscored its role in attenuating lipid dysregulation, exemplified by downregulating Slc25a47, a mitochondrial lipid transporter (41), and activating PPARG, a regulator of fatty acid oxidation, aligning with preclinical evidence of kaempferol reducing aortic plaque burden in murine models (18).

To provide a broader therapeutic perspective, it is essential to contextualize our findings alongside other potential molecules for PAD. Currently, clinical pharmacotherapy relies heavily on antiplatelet agents, statins, and vasodilators like cilostazol, which improves claudication distance but is often limited by side effects such as headaches and palpitations (42). Additionally, anti-proliferative agents like paclitaxel are utilized in drug-coated balloons to prevent restenosis, yet concerns regarding long-term safety remain (43). In the realm of natural compounds, other flavonoids such as quercetin and resveratrol have shown promise in mitigating endothelial dysfunction via antioxidant mechanisms (44). However, distinct from these agents, our systems-level data interpretation reveals that kaempferol uniquely bridges the gap between macrophage polarization and ferroptosis. Unlike standard therapies that primarily target platelet aggregation or general inflammation, kaempferol appears to specifically remodel the immuno-metabolic microenvironment, offering a complementary strategy to address the residual inflammatory risk in PAD.

The expression analysis of kaempferol targets within the PAD microenvironment highlights cell type-specific therapeutic opportunities. High expression of CD36 and PRKCH in ECs implicates these genes in endothelial dysfunction, a hallmark of PAD pathogenesis. CD36, a scavenger receptor, facilitates oxidized LDL uptake and promotes pro-inflammatory signaling via NF-κB and MAPK pathways, thereby exacerbating endothelial apoptosis and vascular dysfunction (45). Conversely, SYK and NLRC5 in macrophages suggest dual roles in inflammation: SYK drives phagocytosis and cytokine release, while NLRC5 may attenuate NF-κB activation to limit excessive inflammation, as demonstrated in cardiac injury models (46, 47). Moreover, kaempferol’s anti-inflammatory efficacy was further validated through its suppression of LPS-induced M1 polarization and NO overproduction. These findings collectively position kaempferol as a multi-target agent capable of addressing endothelial dysfunction and macrophage-driven inflammation in PAD.

We acknowledge certain limitations in our in vitro validation scope. While the murine RAW264.7 line serves as a robust, universally accepted gold-standard model for screening core highly-conserved anti-inflammatory pathways (such as NF-κB and NRF2), future studies utilizing human primary monocyte-derived macrophages or THP-1 cells are warranted to fully bridge the cross-species gap. Additionally, while our bioinformatic analysis highlighted the prominence of CD36 in the PAD macroenvironment, our experimental phase utilized LPS rather than oxidized LDL. We deliberately utilized LPS to model the acute, severe inflammatory exacerbations and TLR4-mediated endotoxemic microenvironments frequently complicating advanced ischemic PAD and diabetic ulcerations. Given the established crosstalk wherein LPS-induced TLR4 activation primes macrophages for enhanced lipid uptake via scavenger receptors, our model validates the baseline hyper-inflammatory attenuation. Although we have validated the pro-angiogenic effect of kaempferol in zebrafish VRI model, mammalian hindlimb ischemia PAD animal models are still required to further translate our findings to clinical-relevant disease phenotypes.

In conclusion, this study transcends the traditional understanding of kaempferol’s cardiovascular benefits by utilizing a comprehensive multi-omics framework. We proposed a dual-track parallel mechanism: kaempferol acts on high-inflammatory macrophages in PAD lesions to independently suppress inflammatory signaling and alleviate ferroptotic stress, and the improved ischemic microenvironment ultimately promotes vascular repair verified by zebrafish angiogenesis assay. We successfully identified the crosstalk between macrophage polarization and ferroptosis as the key mechanism underlying kaempferol’s therapeutic efficacy in PAD.

## Data Availability

The original contributions presented in the study are included in the article/**Supplementary Material**. Further inquiries can be directed to the corresponding authors.
