# Supplementary material for: Multi-omics integration reveals macrophage polarization and ferroptosis as key mechanisms underlying kaempferol’s therapeutic efficacy in peripheral artery disease
Source: Front Immunol. 2026 Sep 3;17:1733284. doi: 10.3389/fimmu.2026.1733284 (PMC13582352; doi:10.3389/fimmu.2026.1733284)
Supplement: Supplementary file 1 [file Table1.docx]

| Gene | Primer sequence (5′–3′) |
| --- | --- |
| IL6(F) | CCACTTCACAAGTCGGAGGCTTA |
| IL6(R) | TGCAAGTGCATCATCGTTGTTC |
| IL-1β(F) | TGGACCTTCCAGATGAGGACA |
| IL-1β(R) | GTTCATCTCGGAGCCTGTAGTG |
| iNOS(F) | ACAACAGGAACCTACCAGCTCA |
| iNOS(R) | GATGTTGTAGCGCCTGTGTGTCA |
| TNF-α(F) | GAGCACAGAAAGCATGATCCG |
| TNF-α(R) | TAGACAGAAGAGCGTGGTGG |
| GAPDH(F) | AAATGGTGAAGGTCGGTGTG |
| GAPDH(R) | AAATGGTGAAGGTCGGTGTG |

Supplementary Table 1: Primer sequences for qPCR.

Supplementary Table 2: Molecular docking parameters and binding affinities (Vina scores) of kaempferol (KAE) and native ligand controls (CTRL) with key targets.

| Term | Vina score | Cavity volume (Å3) | Center (x, y, z) | Docking size (x, y, z) |
| --- | --- | --- | --- | --- |
| TNF-KAE | -8.9 | 1513 | 55, -7, 9 | 21, 21, 21 |
| TNF-CTRL | -13.6 | 1513 | 55, -7, 9 | 27, 27, 27 |
| SYK-KAE | -7.9 | 1165 | 4, 2, 10 | 21, 21, 21 |
| SYK-CTRL | -8.5 | 1165 | 4, 2, 10 | 26, 26, 26 |
| LCK-KAE | -8.4 | 408 | 23, 38, 84 | 21, 21, 21 |
| LCK-CTRL | -9.5 | 408 | 23, 38, 84 | 24, 24, 24 |
| RTP4-KAE | -7.8 | 267 | 11, -1, -11 | 21, 21, 21 |
| IL4I1-KAE | -9.2 | 2481 | 1, -2, 0 | 21, 33, 35 |
| HHEX-KAE | -6.4 | 219 | 1, 7, -1 | 21, 21, 21 |
| KCNE3-KAE | -5.4 | 76 | -19, 2, 32 | 21, 21, 21 |
| SNX5-KAE | -7.7 | 1490 | 0, 25, -22 | 21, 21, 21 |
